# Identification and Genome-Wide Gene Expression Perturbation of a Trisomy in Chinese Kale (*Brassica oleracea* var. *alboglabra*)

**DOI:** 10.3390/plants12183199

**Published:** 2023-09-07

**Authors:** Qun Feng, Junxing Yu, Jie Yu, Mingyang Hu, Lei Gu, Hongcheng Wang, Xuye Du, Bin Zhu, Mengxian Cai

**Affiliations:** School of Life Sciences, Guizhou Normal University, Guiyang 550025, China; 21010100399@gznu.edu.cn (Q.F.); yujunxing@gznu.edu.cn (J.Y.); 21010100402@gznu.edu.cn (J.Y.); 222100100386@gznu.edu.cn (M.H.); 201808009@gznu.edu.cn (L.G.); wanghc@gznu.edu.cn (H.W.); duxuye@gznu.edu.cn (X.D.)

**Keywords:** trisomy, *Brassica oleracea*, RNA-seq, gene expression perturbation

## Abstract

Trisomy harbouring an extra copy of the chromosome generally causes a variety of physical and intellectual disabilities in mammals but is an extremely rare and important genetic stock in plants. In this study, a spontaneous trisomy plant in a Chinese kale accession (*Brassica oleracea* var. *alboglabra*, CC, 2*n* = 18) that showed significantly smaller plant architecture when compared to other normal plants was found and subsequently confirmed by cytological analysis in which the chromosome set of 2*n* = 19 and abnormal chromosome behaviour were observed. Then, based on the gene expression deviation determined by RNA-seq, the extra chromosome copy in this trisomy was identified as chromosome C2 (TC2). Compared to normal plants, TC2 not only showed generally upregulated differentially expressed genes (DEGs) on chromosome C2 (97.21% of 573 DEGs in chromosome C2) but also exhibited a whole-genome expression perturbation, in which 1329 DEGs (69.87% of total DEGs) were observed along two-copy chromosomes (*trans*-effect). The genes in the high (gene expression value > 100) and medium (100 > gene expression value > 10) groups were more prone to decreased gene expression, but the genes in the low group (10 > gene expression value > 0.1) showed upregulated expression deviation. In addition, GO (Gene ontology) annotation analysis revealed that the upregulated DEGs in the *trans*-effect group were overrepresented by the genes involved in the response to stress category, while the downregulated DEGs in the *trans*-effect group were mostly enriched in pathways related to DNA synthesis. In conclusion, we think our results can provide important resources for genetic analysis in *B. oleracea* and show some novel insights for understanding trisomy plant biology.

## 1. Introduction

An aneuploid is an organism with an abnormal number of chromosomes that can occur due to errors in cell division during gametogenesis or early embryonic development, resulting in a loss or gain of one or more chromosomes [1,2,3]. Trisomy, as a common type of aneuploidy, is a genetic stock in which an individual has three copies of a particular chromosome instead of the normal two [4]. The extra chromosome generally results in an imbalance of chromosome material that can cause a variety of physical and intellectual disabilities. Therefore, trisomy with extra chromosomes generally shows delayed growth patterns, infertility, and other developmental abnormalities. In humans, some examples of trisomy disorders include Down syndrome (trisomy 21), Edwards syndrome (trisomy 18), and Patau syndrome (trisomy 13). Plants have a higher tolerance to these aneuploids than animals [5]. Based on different types of extra chromosomes, trisomy can be divided into primary, secondary, tertiary, and terminal trisomy in plants [6]. These trisomy plants can be advantageous to deciphering the gene dosage, *trans*-effect on gene expression, and the function of certain genes on these variant chromosomes. In particular, primary trisomy plants can be used to locate traits and linkage groups to extra chromosomes [7,8], which can be used as an important genetic stock to construct a physical map of genes [9,10,11]. In addition, researchers believe that trisomy in plants can also have implications for genetic diversity because the trisomic individuals in a population can add genetic variation, which can increase the adaptability and resilience of the population in changing environments [12,13,14].

A century ago, Blakeslee (1922) [15] first reported a complete set of primary trisomies in Jimson weed (*Datura stramonium* L., 2*n* = 24), in which the 12 trisomic plants showed obvious morphological differences from the diploid parent. After that, the trisomy plants were distinguished in many different plants, including maize (*Zea mays* L.) [16], *Gossypium hirsutum* L. [17], broad bean (*Vicia faba* L.) [18], rice (*Oryza sativa* L.) [14], *Plantago lagopus* L. [19], and *Populus* [6]. Seeds that were exposed to γ-rays have been reported to obtain trisomy in *G. hirsutum* L. [17]. Microspore culture is believed to be an effective method to generate different trisomy plantlets in some crops [20,21]. In addition, crosses between triploid and diploid species, which are attributed to the irregular segregation of chromosomes at meiosis in triploid parents, have been recognized as a purposeful method to obtain trisomy plants and have been successfully utilized in tomato (*Solanum lycopersicum* L.) [22], *Z. mays* L. [23], rice [24], *P. lagopus* L. [25], and *Populus* [6]. In addition, as trisomy in *D. stramonium* L., some trisomy plants which are not prone to take place can be identified in natural variations [26].

Recently, studies have reported that aneuploidy, including trisomy in plants, can affect gene expression that includes not only the genes on the extra chromosome (*cis*-effects) but also the genes on other normal chromosomes (*trans*-effects), leading to abundant changes in their phenotype [14,27,28,29,30,31]. Compared to the variations in ploidy, aneuploidy plants, including trisomy, generally produced much greater modulations of global gene expression [14,30,32,33]. Studies have shown that, compared to *cis*-acting effects on aneuploid chromosomes, *trans*-acting effects are quite prevalent in the majority of aneuploid organisms [34,35,36]; however, an exception was observed in yeast, in which very few *trans*-effects were observed in aneuploid yeast [37]. Recently, the work of Sun et al. [14] showed that trisomies in rice caused not only immediate *trans*-effects on gene expression in trisomy per se but also far-reaching effects on gene expression in their normal offspring.

As a species in the triangle of U [38], *Brassica oleracea* (CC, 2*n* = 18), which comprises many cultivars, such as cabbage, kalian, broccoli, cauliflower, kale, and collard greens, has been widely used as an important vegetable because it is rich in a variety of essential nutrients [38,39,40,41]. Intriguingly, a high frequency of trisomy was observed in the offspring of diploid progenitors in cauliflower [42], which resulted in considerable economic loss. However, to our knowledge, trisomy plants have not been observed in other cultivars of *B. oleracea*. In this study, a trisomy that was spontaneously generated in Kalian was detected. To identify the trisomy type and decipher the gene expression profile in this trisomy, RNA-seq was carried out. The results of RNA sequencing showed that the extra chromosome was C2. In addition, compared to diploid plants, a widespread *trans*-effect that was obviously overrepresented by upregulated DEGs associated with “response to stress” and by downregulated DEGs involved in “DNA synthesis” was observed. We believe that the trisomy in Chinese kale provides important resources for genetic analysis in *B. oleracea* and provides some insights for understanding trisomy plant biology.

## 2. Results

### 2.1. Cytological Analysis and Characteristics of the Trisomy Plant

**Figure 1:**
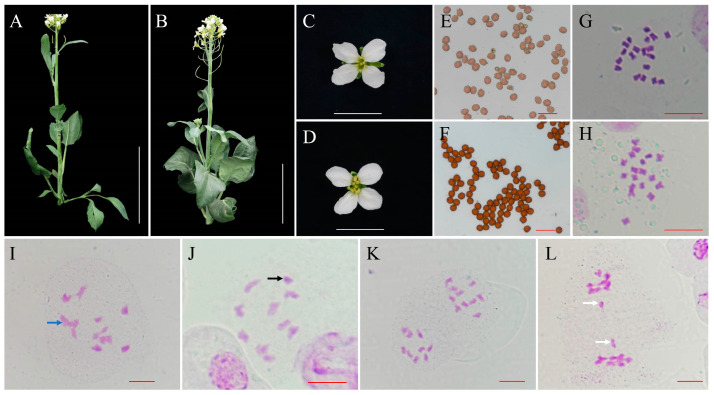
Morphological analysis of the trisomy and normal *B. oleracea* plants. (**A**,**B**) Plant architectures of trisomy and normal *B. oleracea* plants at the flowering stage. Bar: 15 cm. (**C**,**D**) Flower phenotypes of trisomy and normal *B. oleracea* plants. Bar: 2 cm. (**E**,**F**) Pollen grain stainability of trisomy and normal *B. oleracea* plants. Bar 50 μm. (**G**,**H**) The chromosome number of trisomy (2*n* = 19) and normal plants (2*n* = 18). Bar: 10 μm. (**I**,**J**) PMCs of trisomy at diakinesis showed a dominant chromosome pair configuration of eight bivalents and a trivalent (blue arrow) and nine bivalents and a univalent (black arrow). Bar: 10 μm. (**K**,**L**) PMCs of trisomy at anaphase I showed chromosome segregation of 9:10, and lagging chromosomes (white arrows) exhibiting segregation of 9:9 were observed. Bar: 10 μm.

In the spring of 2023, we found that a Chinese kale “Chijielan” plant showed obviously smaller architecture, smaller and vimineous leaves, and delayed flower time compared to other plants in the experimental field (Figure 1A,B). The flower of the variant plant (Figure 1C) was similar to that of these sister lines but showed weakened stamens (Figure 1D). The pollen staining ability of the variant plants (87.63% ± 1.24% and 96.57% ± 2.42% in variant and normal plants, respectively) was extremely significantly lower than that of normal plants (*t* test, *p* < 0.01) (Figure 1E,F). After checking the chromosome number of the variant plant, this plant had a chromosome set of 2*n* = 19 (Figure 1G), which showed an extra copy of a chromosome compared to the normal plant (2*n* = 18, Figure 1H), suggesting that this variant plant should be a trisomy. The explants from the trisomy were used to generate cloned plantlets using MS medium with 6-BA and NAA for further analysis.

Then, we checked the chromosome behaviour of the trisomy in meiosis. A total of 67 pollen mother cells (PMCs) with trisomy at diakinesis (DI) were observed and analysed. Among these PMCs, 62.69% (42 PMCs) showed a dominant chromosome pair configuration (Figure 1I) of eight bivalents and a trivalent, whereas a small portion of PMCs (25 PMCs) had a configuration of nine bivalents and a univalent (Figure 1J). In addition, 72 PMCs of trisomy at anaphase I (AI) were observed. The majority of the PMCs (65 PMCs, 90.28%) at AI showed chromosome segregation ratios of 9:10 (Figure 1K). Only 9.72% of PMCs (7 PMCs) with lagging chromosomes exhibiting segregation of 9:9 were observed (Figure 1L).

### 2.2. Determination of the Genotype of the Trisomy Plant Using RNA-Seq

**Figure 2:**
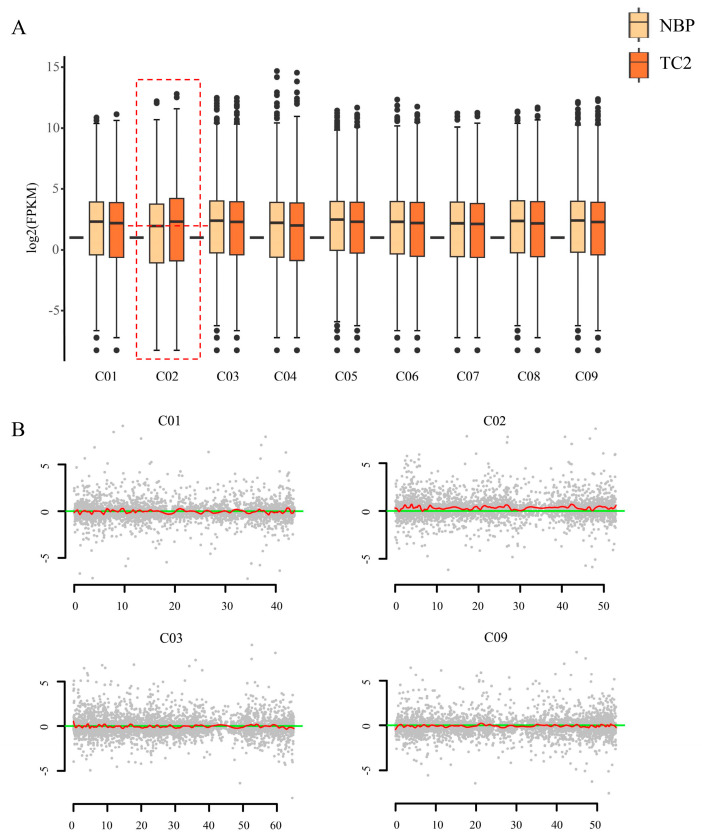
Determination of the extra chromosome in trisomy. (**A**) Box plot of log_2_(FPKM) values of the total expressed genes (FPKM > 0) located on each of chromosomes in NBP and TC2. Chromosome C2 in trisomy showed a significantly high median value of gene expression (dashed box). (**B**) Gene expression analysis performed on each of chromosomes to measure the expression deviation. Red lines denote the smoothed distribution for the differentially expressed genes in these selected chromosomes. The *y* axis represents the log2(fold change) value of FPKM between the NBP and TC2. The *x* axis represents the sorted positions of genes on these chromosomes. Taking chromosomes C01, C02, C03, and C09 as examples.

RNA-seq, which has been used to calculate gene expression profiles, has been demonstrated as a powerful method for determining the genotype of aneuploidy [30,32,43]. In this study, 41.0–65.4 million clean reads were obtained for each replicate with Q30 values of 95.12–95.46% after removing the adapters and trimming the low-quality reads. In addition, 93.09–93.71% clean reads were mapped to the reference genome (Table S1). To determine the extra chromosome in trisomy, the log_2_ values (FPKM values) of all expressed genes along all nine chromosomes were exhibited using box plots. We noticed that obviously increased gene expression was observed on chromosome C2 in the trisomy when compared to normal plants (Figure 2A), indicating that the extra chromosome is likely C2. To further confirm the result, the distribution of gene expression fold changes (log2-fold change) along each normal chromosome between trisomy and normal plants was determined and smoothed to measure the expression deviation (Figure 2B). The results showed that the genes along chromosome C2 were generally highly expressed, confirming that the trisomy genotype was Trisomy C2, abbreviated as TC2 in this study.

### 2.3. DEGs Determination in Trisomy Plants and qRT–PCR Validation

**Figure 3:**
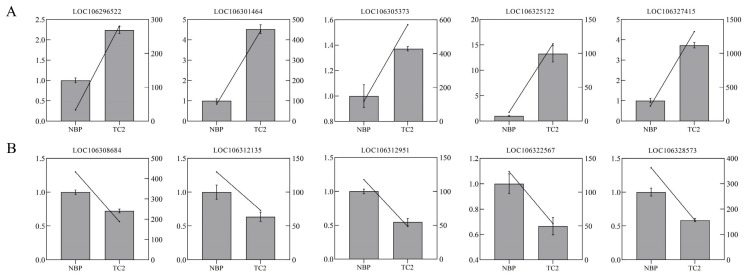
qRT–PCR verification of the expression levels of 10 randomly selected DEGs. (**A**) The expression levels of five randomly selected up-regulated genes. (**B**) The expression levels of five down-regulated genes were randomly selected. The left *y*-axis represents the relative expression level, and the right *y*-axis represents the results of RNA-seq (FPKM). The box with error bars indicates the result of qRT–PCR, and the oblique line represents the FPKM value of gene expression.

The false discovery rate (FDR) with q < 0.05 and a 1.5 fold change in gene expression were used as a cut-off to determine DEGs in this study, taking into consideration that chromosome C2 in TC2 had a 1.5 fold change in normal *B. oleracea* plants (abbreviated as NBP in the RNA-seq analysis). In total, 1902 DEGs, comprising 1219 upregulated and 683 downregulated DEGs, were detected in the comparison of NBP vs. TC2. Then, qRTPCR validation of ten selected DEGs was used to confirm the accuracy of RNA-seq, and the results were consistent with those of RNA-seq (Figure 3). The actin gene (LOC106295461) was used as an internal reference, which was stably expressed both in NBP and TC2 (Figure S1).

### 2.4. Cis- and Trans-Effect DEGs in TC2

Among these DEGs, a total of 573 DEGs, accounting for less than one-third (30.13%) of the total DEGs, were caused by *cis*-effects, which were attributed to the extra chromosome C2 (Table 1). In addition, the expression level of the majority (557 DEGs, 97.21% of *cis*-effect DEGs) of the *cis*-effect DEGs responded to this copy number change, suggesting that these genes showed dose effects. However, 16 DEGs along variant chromosome C2 were downregulated, showing the reverse gene dosage. Additionally, we noticed that TC2 exhibited prevailing *trans*-effect DEGs after trimming the DEGs along chromosome C2, in which approximately 70% of the total DEGs (1329 DEGs) were along the whole genome. Among these trans-effect DEGs, 667 downregulated DEGs (50.19%) and comparable upregulated DEGs (662 DEGs, 49.81%) were detected (χ^2^-test, *p* > 0.05).

Then, we computed the proportion of *trans*-effect DEGs in the total genes of each of the normal chromosomes to detect whether *trans*-effect DEGs randomly or partially affected each chromosome of aneuploidy in TC2. The results (Table 2) showed that 116–224 DEGs per chromosome, occupying 2.15–3.54% of the total genes of each of the chromosomes, were observed. Briefly, chromosome C1 gave rise to the highest proportion of DEGs (179 DEGs, 3.54%), followed by comparable C7 (191 DEGs, 3.51%), C4 (185 DEGs, 3.09%), and C9 (188 DEGs, 3.02%) (χ^2^-test, *p* > 0.05). Chromosomes C3 (224 DEGs, 2.79%), C8 (127 DEGs, 2.41%), and C5 (119 DEGs, 2.15%) showed relatively few DEGs, indicating that these chromosomes were less likely to be affected in TC2. Moreover, the upregulated and downregulated DEGs along each chromosome were comparable (χ^2^-test, *p* > 0.05), except on chromosomes C1 and C4, in which dominant downregulated DEGs were detected (χ^2^-test, *p* < 0.05).

**Figure 4:**
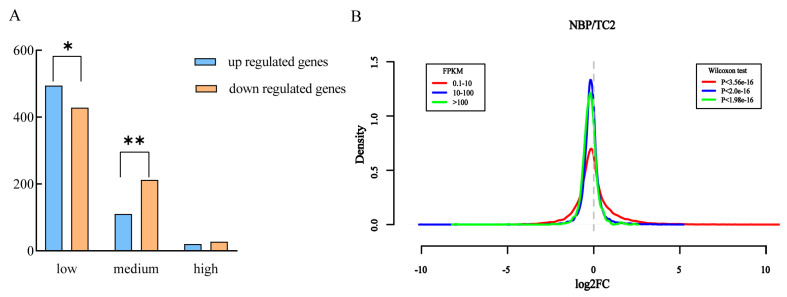
Impact of extra chromosomes on gene expression changes in different gene expression groups. (**A**) The gene numbers of different expression groups and *trans*-effect DEGs in different gene expression groups. “*” and “**” indicate that the DEG groups were significantly different at the levels of *p* < 0.05 and *p* < 0.01 (χ^2^ test). (**B**) The density of the frequency distribution of log2 (fold change) in NBP vs. TC2 showed that different gene groups exhibited inconsistent response patterns to extra chromosome C2. The *x*-axis represents the log2 (fold-change) in gene expression levels between the NBP and euploid TC2 cells in three gene groups, and the *y*-axis represents the frequency distribution of log2 (fold-change) (Wilcoxon test, q < 0.05).

According to the work of Zhu et al. [32], we divided the genes along normal chromosomes into low (0.1 < FPKM < 10), medium (10 < FPKM < 100), and high (FPKM > 100) groups based on the gene expression values in normal *B. oleracea*. In total, 21,428, 9433, and 1269 genes could be classified into low, medium, and high groups, respectively (Table 3). In addition, 922, 322, and 47 DEGs that were not significantly different were detected among these groups. Intriguingly, we noticed that upregulated DEGs were significantly higher than downregulated DEGs in the low group (χ^2^ test, *p* < 0.05), whereas the downregulated DEGs were extremely dominant in the medium group (χ^2^ test, *p* < 0.01), and the downregulated DEGs were higher than upregulated DEGs but not significant in the high group (Table 3; Figure 4A). Then, the whole gene expression fold-change density of NBP vs. TC2 for all groups was determined to check whether the different gene groups showed biased gene expression. The results (Figure 4B) showed that genes in the low group were prone to high expression in the comparison of NBP vs. TC2, whereas genes in the medium and high groups were biased towards downregulation.

### 2.5. GO Analysis of Cis- and Trans-Effect DEGs

**Figure 5:**
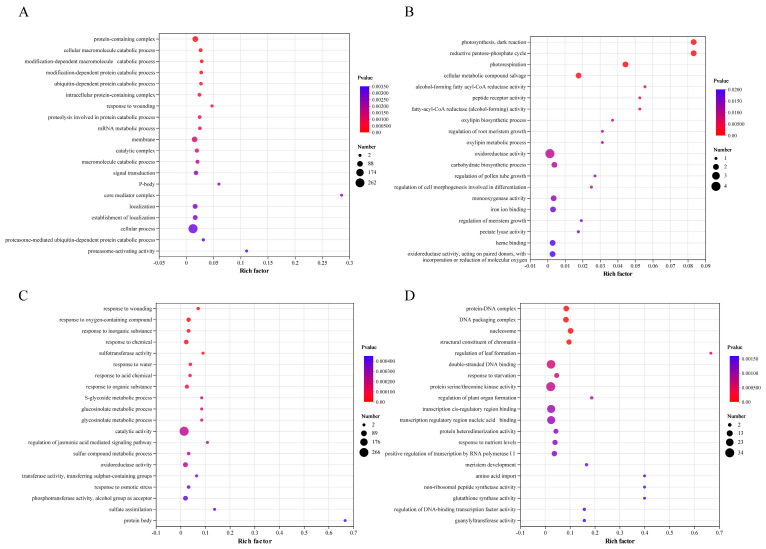
Top 20 GO enrichment functions of *trans*-effect and *cis*-effect DEGs. (**A**,**B**) Top 20 GO enrichment functions enriched by upregulated and downregulated *cis*-effect DEGs, respectively. (**C**,**D**) Top 20 GO enrichment functions overrepresented by upregulated and downregulated *trans*-effect DEGs. The *x*-axis represents the ratio of the number of DEGs (sample number) enriched (based on the rich factor) in a term to the number of annotated DEGs (background number), and the *y*-axis represents the term name.

Based on the GO annotation analysis (Figure 5), the upregulated DEGs caused by the *cis*-effect were mainly involved in functions related to catabolic processes (Figure 5A), such as “protein-containing complex”, “cellular macromolecule catabolic process”, “modification-dependent macromolecule catabolic process”, and “modification-dependent protein catabolic process”. The few downregulated DEGs caused by the *cis*-effect were mainly involved in “photosynthesis, dark reaction”, “reductive pentose-phosphate cycle”, and “photorespiration” (Figure 5B), suggesting that these downregulated DEGs were related to photosynthesis. Moreover, the upregulated DEGs in the *trans*-effect group were over-represented by the genes involved in response to stress (Figure 5C), such as “response to wounding”, “response to oxygen-containing compound”, “response to inorganic substance”, and “response to chemical”. The downregulated DEGs in the *trans*-effect group were mainly involved in “protein–DNA complex”, “DNA packaging complex”, “nucleosome”, and “structural constituent of chromatin” (Figure 5D), indicating that significantly impaired synthesis of DNA was caused by aneuploidy in TC2.

## 3. Discussion

Trisomic plants generally show a deficient phenotype but can be advantageous to genetic diversity because the trisomic individuals in a population can add genetic variations that likely increase the adaptability and resilience of the population in changing environments [1,3,6,12,32,42]. Recently, researchers detected a high frequency of trisomy in cauliflower, a cultivar variant of *B. oleracea*; however, the types of these trisomies have not been further identified [42]. Herein, a trisomic plant in Kalian was occasionally obtained, which was subsequently distinguished as trisomy C2 by RNA-seq results. RNA-seq, used to assess gene expression profiles along variant chromosomes, has been demonstrated to be an effective method to identify the type of aneuploidy [14,32,42,43] because the majority of gene expression profiles along variant chromosomes show changes in accordance with copy number changes in chromosomes. With the RNA-seq data, a nullisomic in *B. napus* [30] and several trisomies in rice [14] were successfully determined. Moreover, trisomic variations are generally homogeneous, resulting in challenges in developing molecular markers to identify trisomy.

Viable aneuploidy generally shows a severely deficient phenotype, likely attributed to disruption of the stoichiometric regulatory network of gene expression [5,30,42,44]. Only trisomy 19 in mice, in which the extra chromosome is the smallest autosome, can evade embryonic lethality [45]. In humans, Down’s syndrome, which is caused by the whole or part of an extra copy of chromosome 21, the smallest autosome, is the most common chromosomal abnormality and the only chromosome syndrome which allows those affected to survive into adulthood [46,47,48]. It is likely that the variant chromosome harbouring fewer genes survives more easily in mammals. Plants have better aneuploidy tolerance than mammals [5,30,41]. In this study, the trisomy plant was eventually demonstrated to harbour a third copy of chromosome C2, which is not the smallest chromosome in *B. oleracea*. Intriguingly, a nullisomic of chromosome C2 was obtained in natural *B. napus* during interspecific hybridization between *B. napus* and *Capsella bursa-pastoris* [30,49]. In addition, the work of Xiong et al. [50] showed that more than 50% of the lines in the progeny of resynthesized *B. napus* were aneuploid on chromosome C2. These results indicate that chromosome C2 is likely prone to alteration.

Previous studies have shown that abnormal chromosomes in aneuploid can disrupt the balance of gene expression, causing gene expression perturbation across the whole normal genome (*trans*-effect), thereby reducing the health and viability of the organism [3,6,14,30,31]. Several studies involving gene expression in different aneuploid organisms showed that the *cis*-effect greatly exceeded the *trans*-effect [14,30,31]. This finding can be explained by the hypothesis of genome balance because the strict stoichiometry of all dosage-sensitive genes is disturbed by the altered chromosome in aneuploidy [27,50,51,52]. In this study, a dominant *trans*-effect (approximately 70%) was also observed in trisomic *B. oleracea*, reconfirming the reliability of the hypothesis of genome balance. Moreover, we noticed that the *trans*-effect upregulated DEGs were mainly enriched in the functional terms associated with response to stress. Interestingly, a study involving different aneuploidies of yeast showed a signature characteristic of the Environmental Stress Response (ESR) of gene expression [37]. Similar results were also observed in a recently reported trisomy in rice, in which the upregulated DEGs were reported to mainly associate with various types of responses to stress [14]. Researchers believe that aneuploidy in plants can also have implications for adaptability because aneuploid individuals in a population can add genetic variation, rapidly responding to changing environments [6,14,32,42,53]. The GO results of *trans*-effect upregulated DEGs in this study likely support the possibility of the advantages of trisomy in responding to environmental changes. In addition, different gene expression biases were detected in TC2, in which the genes belonging to the low group were more likely to be upregulated, while genes in the medium and high groups were more likely to be downregulated. Similar results were observed in aneuploidy rice [14], *B. napus* [30], and *B. rapa* [32], and even in Down’s syndrome [46,47,48], suggesting that this gene expression bias should be a signature characteristic of the transcriptional response in aneuploidy.

## 4. Materials and Methods

### 4.1. Plant Materials

A Chinese kale accession, “Chijielan”, which has been self-crossed for more than ten generations, was used in this study. The plants for RNA sequencing were cultured in flowerpots (48 cm in diameter and 30 cm in depth, humus soil, PINDSTRUP, Ryomgård, Denmark) in a greenhouse under a 16:8 light–dark cycle, a stationary temperature of 22 °C, and a relative humidity of 40%. The plants for cytological and phenotypic analyses were cultured in October 2022 in the experimental field of Guizhou Normal University, Guiyang, China for 120 days.

### 4.2. Cytological Analysis

To count the chromosome number of tested plants, young ovaries were collected from trisomic and normal plants and then treated with 2 mM 8-hydroxyquinoline for 3 h in an incubator at a stationary temperature of 22 °C. Then, these ovaries were stored in Carnoy’s solution (3:1 ethanol:glacial acetic) for further analysis. To check the chromosome behaviour of trisomy, the trisomic cloned plantlets were first generated using MS medium with 1.0 mg/L 6-BA (6-benzyladenine) and 0.25 mg/L NAA (naphthylacetic acid), and then young flower buds from trisomic cloned plantlets were collected and continuously treated with fresh Carnoy’s solution until the flower buds were completely discoloured. Cytological analysis was carried out based on the work of Li et al. [54], and the images were captured by a CCD (Charge Coupled Device) camera (N80i, Nikon, Tokyo, Japan).

To check the pollen viability of the tested plants, more than 300 pollen grains from trisomic and normal plants were stained with 1% acetocarmine and then screened using a microscope with a CCD camera. More than three plants for trisomic and normal genotypes were used.

### 4.3. RNA Extraction, c-DNA Library Construction, and RNA Sequencing

For RNA extraction, the third leaves that were newly expanded from trisomic and normal plants were gathered and immediately stored in liquid nitrogen. The total RNA was treated with DNAse (CWBIO, Taizhou, China) to remove DNA contamination. Three biological replicates for trisomic and normal plants were prepared. The leaves were fully ground in a mortar with liquid nitrogen, and approximately 0.1 g of powdered leaves was used to isolate the total RNA using a commercial RNA extraction kit (EASYspin Plant RNA kit, Aidlab, Beijing, China) according to the instructions. Agarose electrophoresis was used to check the quality of the extracted RNA, and an Agilent 2100 instrument (Illumina, San Diego, CA, USA) was used to measure RNA integrity (RIN value). The RIN values of RNA ≥ 8.0 were used to construct the c-DNA library according to the TruSeq RNA Sample Prep v2 protocol (Illumina, San Diego, CA, USA). A total of six c-DNA libraries were constructed and sequenced using the Illumina NovaSeq 6000 platform.

### 4.4. Differentially Expressed Genes (DEGs) Determination

The Illumina sequencing platform generated 150-bp paired-end reads. Then, Trimmomatic version 0.33 was employed to obtain clean reads after removing adapters, poly-N sequences, and low-quality reads (length of reads < 30 bp). These clean reads were mapped to the *B. oleracea* genome [55] using Hisat2 v2.0.5 with default parameters. The FPKM values, which were calculated using RSEM with default parameters (fragments per kilobase of exon per million fragments mapped), were employed to determine gene expression levels. DEGs between the control and treatment *B. oleracea* were determined using R-project based on Benjamini and Hochberg’s approach (cut-off: *p* < 0.05 and fold changes > 1.5). The raw sequence data are available in NCBI-SRA (https://www.ncbi.nlm.nih.gov/sra, accessed on 26 July 2023) under the accession number PRJNA998726.

### 4.5. Real-Time PCR (qRT–PCR) Analysis

To detect the reliability of RNA sequencing, the gene expression levels of the RNA samples were also determined by qRT–PCR. A total of ten DEGs, including five upregulated and five downregulated genes, were randomly selected for qRT–PCR validation. The primers of the selected DEGs and actin (Table S2) were designed by utilizing NCBI Primer-Blast. First-strand cDNA synthesis was performed using a HiFiScript gDNA Removal cDNA Synthesis Kit (CWBIO, Taizhou, China). The actin gene was used as an internal housekeeping gene control. A 10 μL qRT–PCR mixture was prepared by following the protocol for the SYBR-Green fluorescent reagents (TIANGEN Biotech, Beijing, China): 5 μL of 2 × SYBR^®^ Premix Ex Taq II, 0.3 μL each of the forwards and reverse primers, 1 μL (69 ng/μL) of cDNA, and 3.2 μL of RNase-Free ddH_2_O. The qRT-PCR thermal cycling profile consisted of 95 °C for 3 min, followed by 40 cycles of 95 °C for 5 s and 60 °C for 15 s.

## 5. Conclusions

In general, trisomic variation is quite rare in nature. In this study, we found a trisomic plant in *B. oleracea* and subsequently identified the extra chromosome as C2 using cytological analysis and RNA-seq data. Gene expression perturbation across the whole genome was observed in this trisomy (TC2), indicating that the genome balance was severely impacted by the extra chromosome. In addition, GO annotation analysis showed that the downregulated *trans*-effect DEGs were mostly overrepresented by the functions of DNA synthesis. Moreover, the upregulated *trans*-effect DEGs were mainly enriched in functions associated with the response to stress, suggesting the possible advantages of trisomy in responding to environmental changes. We believe that this trisomy not only provides an important resource for genetic analysis in *B. oleracea* but also provides some insights into the regulation and functional interactions of genes in the *B. oleracea* aneuploid genome.

## Figures and Tables

**Table 1:** Summary of *cis*- and *trans*-effect DEGs in the comparison of NBP vs. TC2.

DEGs Groups	Up DEGs (%)	Down DEGs (%)	Total (%)
*cis*-effect DEGs	557 (97.21)	16 (2.79)	573 (30.13)
*trans*-effect DEGs	662 (49.81)	667 (50.19)	1329 (69.87)

**Table 2:** Detailed information on *trans*-effected dysregulated genes along each of the normal chromosomes in the comparison of NBP vs. TC2.

Chromosome	Total Genes	DEGs	Ratio (%)	Up DEGs	Ratio (%)	Down DEGs	Ratio (%)
C1	5056	179 ^a^	3.54	108	60.34	71 **	39.66
C3	8016	224 ^b^	2.79	116	51.79	108	48.21
C4	5996	185 ^ab^	3.09	78	42.16	107 *	57.84
C5	5538	119 ^c^	2.15	62	52.10	57	47.90
C6	4413	116 ^bc^	2.74	66	54.55	50	45.45
C7	5441	191 ^a^	3.51	83	43.46	108	56.54
C8	5272	127 ^bc^	2.41	65	51.18	62	48.82
C9	6222	188 ^ab^	3.02	84	44.68	104	55.32

^a,b,c^ Different groups were calculated by *chi*-square (*p* < 0.05). The group of up-/down-DEGs is significantly higher than oppositely regulated genes (χ^2^, * *p* < 0.05, ** *p* < 0.01).

**Table 3:** The ratio of DEGs distributed in differentially expressed gene level groups.

Gene Expression Groups	EGs	DEGs	Ratio	Up DEGs	Down DEGs
Low	21,428	922	4.30%	494 *	428
Medium	9433	322	3.41%	110	212 **
High	1269	47	3.70%	20	27

The symbols “*” and “**” indicate that the DEG groups were significantly different at the levels of *p* < 0.05 and *p* < 0.01 using the chi-square test.

## Data Availability

Not applicable.

## Supplementary Materials

The following supporting information can be downloaded at: https://www.mdpi.com/article/10.3390/plants12183199/s1, Figure S1: qRT–PCR results of actin gene. Table S1: Summary of clean reads of TC2 and NBP aligned to the reference genome (*B. oleracea*); Table S2: Primers of selected DEGs used in verification of qRT-PCR.
