# Supplementary figures and images for: Identification and Genome-Wide Gene Expression Perturbation of a Trisomy in Chinese Kale (Brassica oleracea var. alboglabra)
Source: Plants (Basel). 2023 Sep 7;12(18):3199. doi: 10.3390/plants12183199 (PMC10536521; doi:10.3390/plants12183199)

A

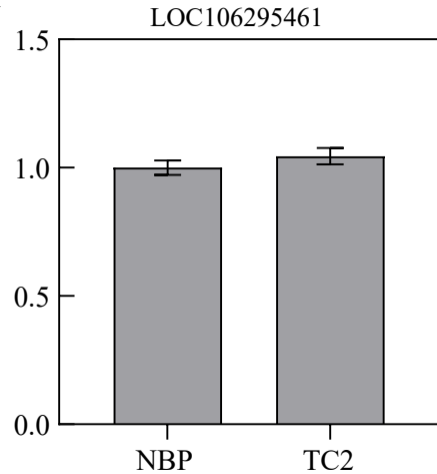

B

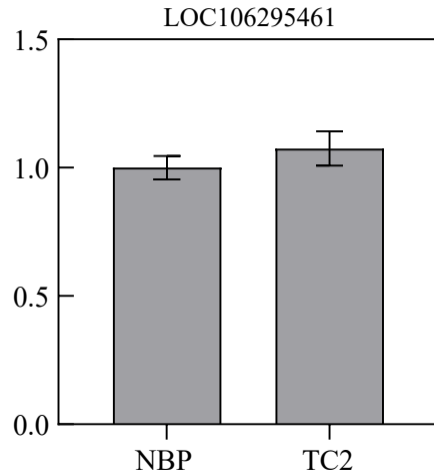

C

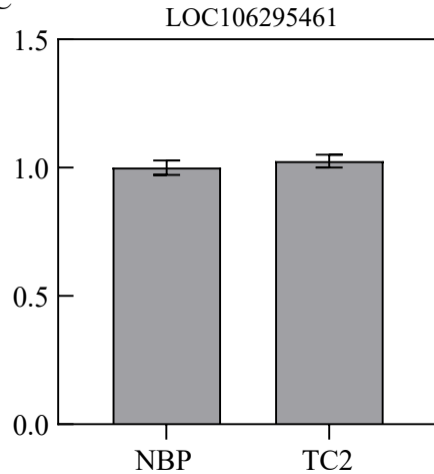

Supplement: Supplementary file 1 [file plants-12-03199-s001.zip › Figure S1.pdf]
